# Supplementary material for: Targeted Therapy for EWS-FLI1 in Ewing Sarcoma
Source: Cancers (Basel). 2023 Aug 9;15(16):4035. doi: 10.3390/cancers15164035 (PMC10452796; doi:10.3390/cancers15164035)
Supplement: Supplementary file 1 [file cancers-15-04035-s001.zip › cancers-2447990-supplementary.pptx]

## Slide 1
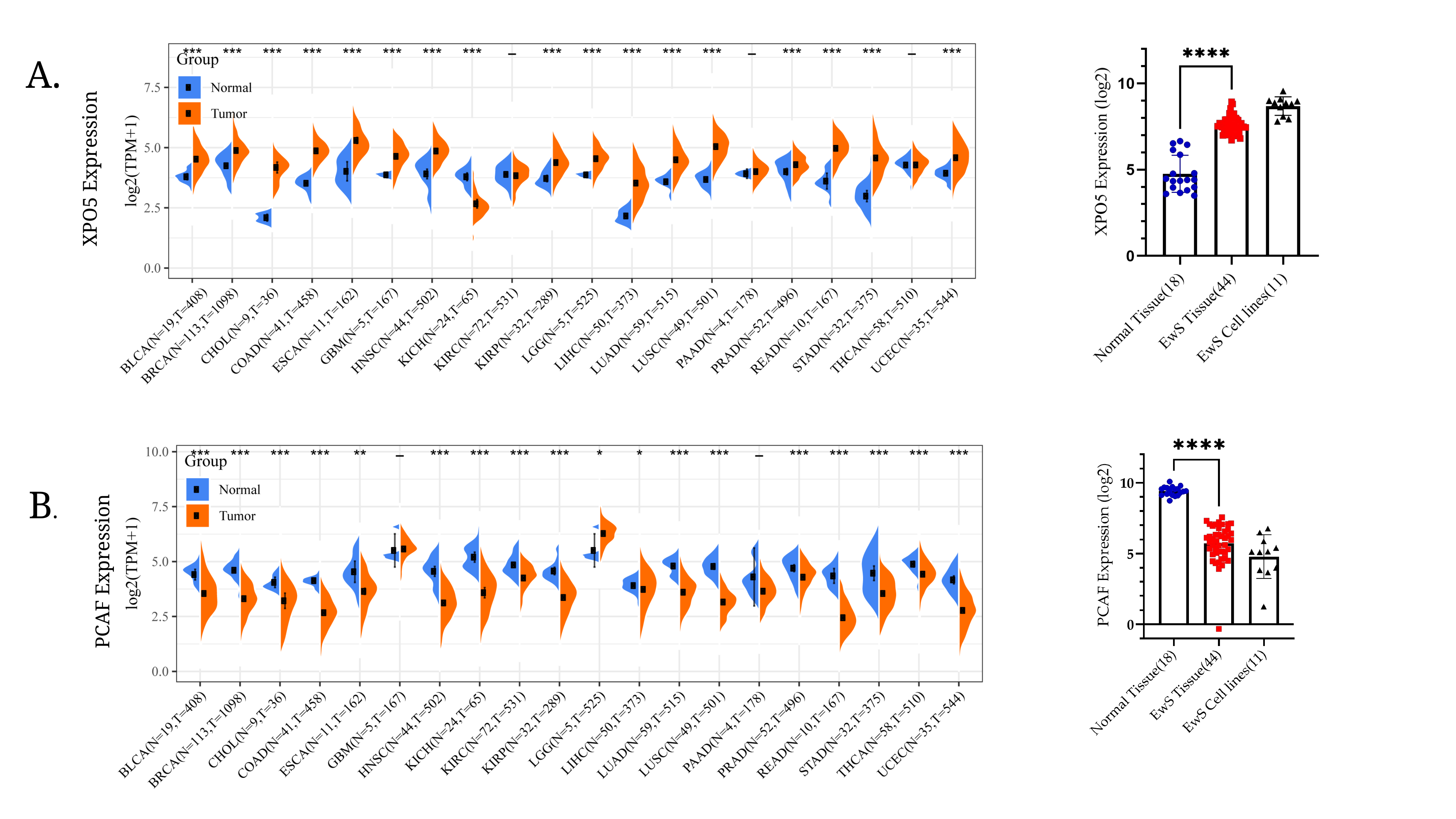

A.
XPO5 Expression
B.
PCAF Expression

## Slide 2
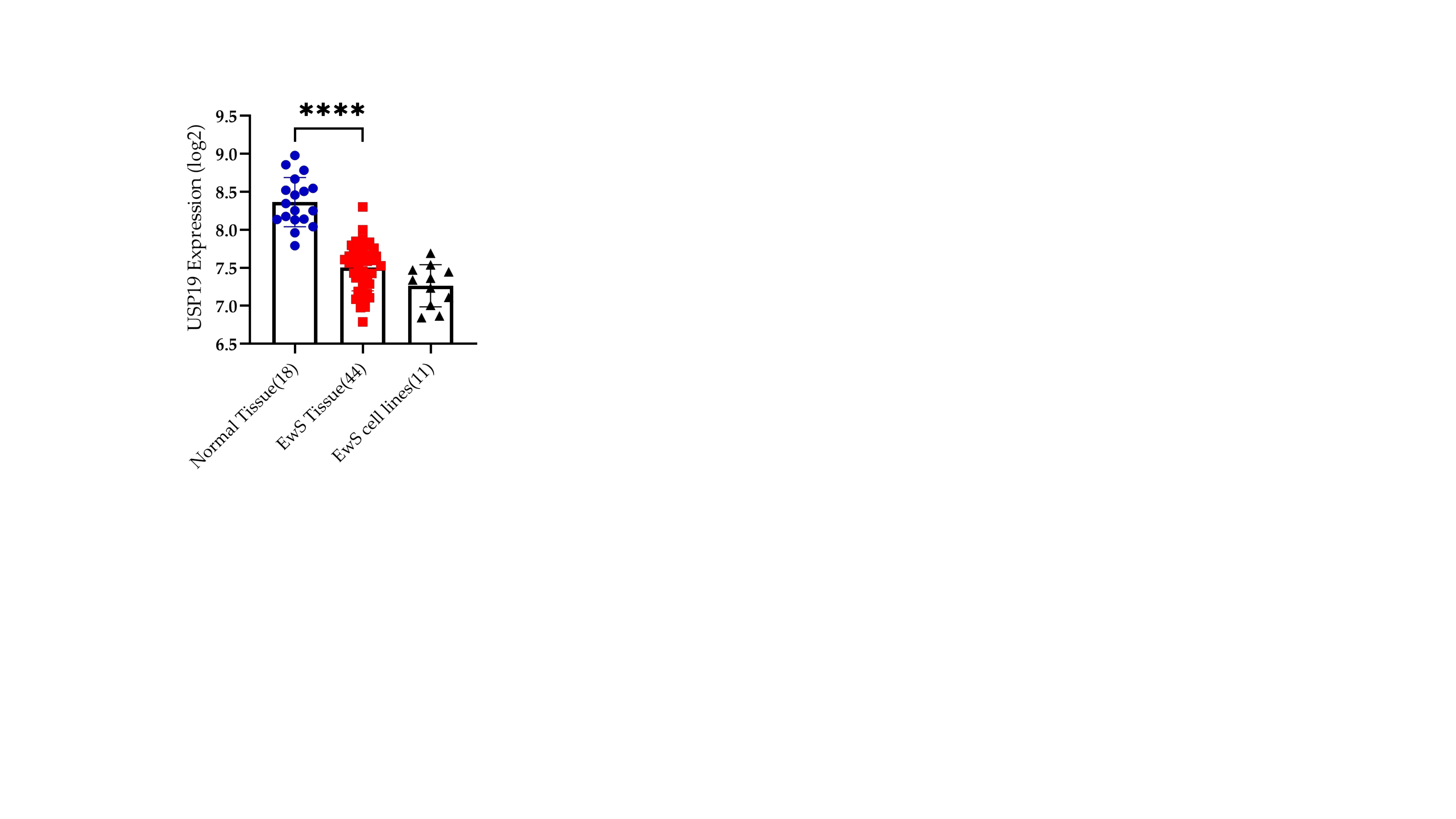

## Slide 3
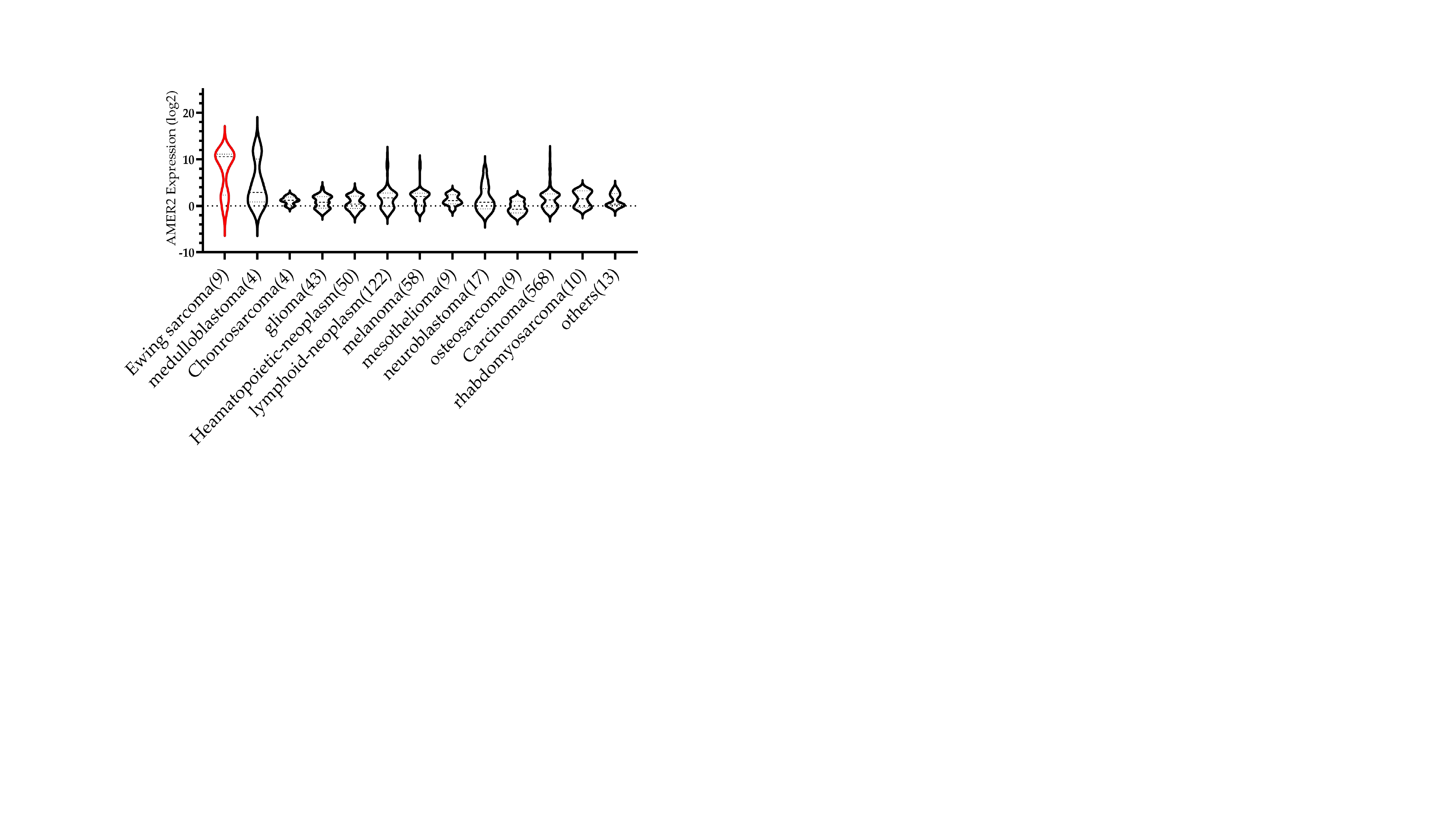

## Slide 4
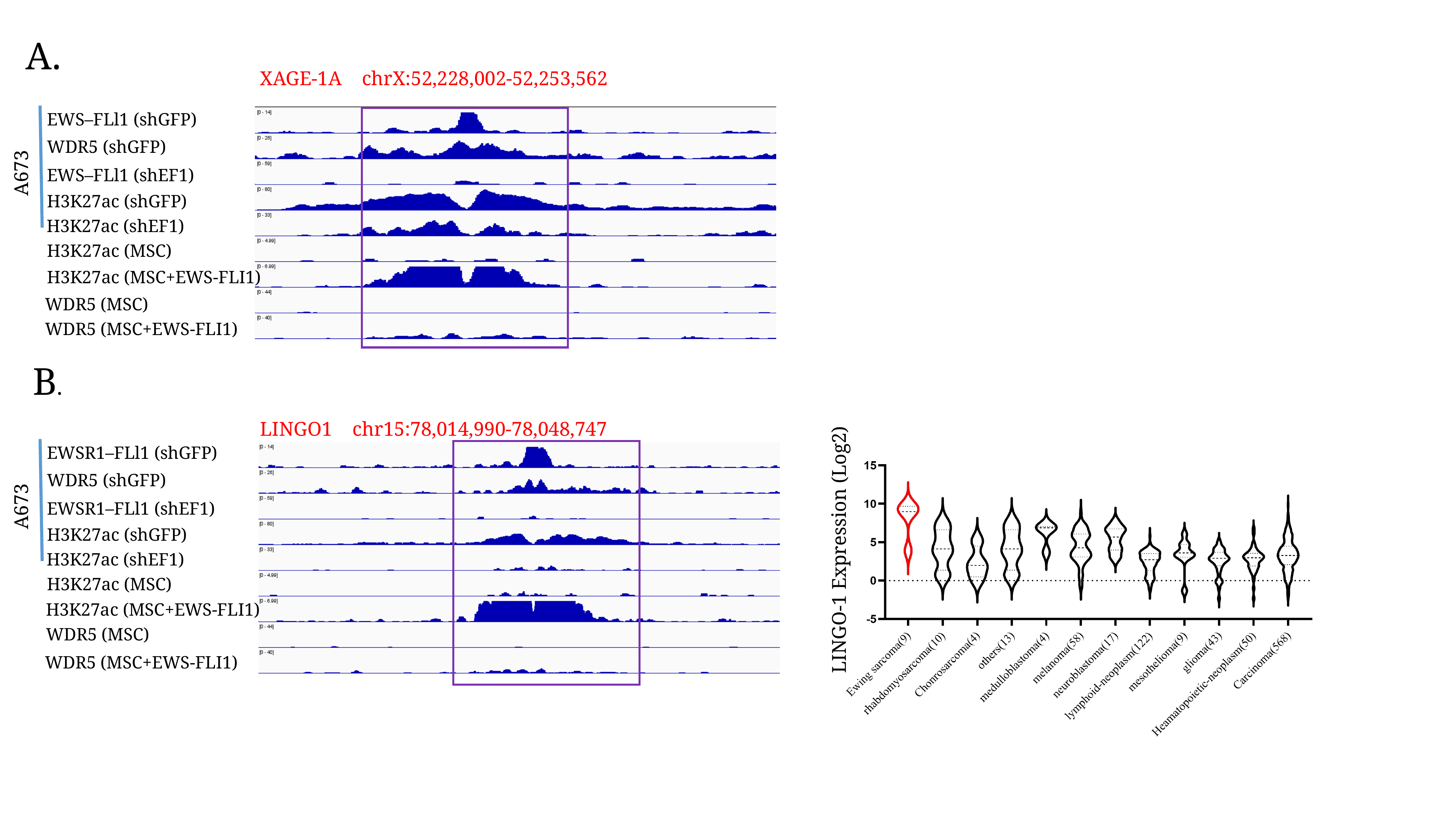

A.
XAGE-1A chrX:52,228,002-52,253,562
EWS–FLl1 (shGFP)
A673
WDR5 (shGFP)
EWS–FLl1 (shEF1)
H3K27ac (shGFP)
H3K27ac (shEF1)
H3K27ac (MSC)
H3K27ac (MSC+EWS-FLI1)
WDR5 (MSC)
WDR5 (MSC+EWS-FLI1)
B.
LINGO1 chr15:78,014,990-78,048,747
EWSR1–FLl1 (shGFP)
A673
WDR5 (shGFP)
EWSR1–FLl1 (shEF1)
H3K27ac (shGFP)
LINGO-1 Expression (Log2)
H3K27ac (shEF1)
H3K27ac (MSC)
H3K27ac (MSC+EWS-FLI1)
WDR5 (MSC)
WDR5 (MSC+EWS-FLI1)
